# Online Behavioral Digital Phenotyping for Depression: Scoping Review of Behavioral Features and Exploratory Assessment of Research Domain Criteria Construct Relevance

**DOI:** 10.2196/101329

**Published:** 2026-10-21

**Authors:** Qiuyan Ren, Jing Chi, Yuman Li, Ping Zhao, Meiqin Luo, Jiale Sun, Yan He, Jianhong Liu, Lin Luo, Yanni Wu

**Affiliations:** 1 Nanfang Hospital Southern Medical University Guangzhou China; 2 School of Nursing Southern Medical University Guangzhou China; 3 The Second Xiangya Hospital Central South University Changsha China

**Keywords:** digital phenotyping, behavioral markers, RDoC, research domain criteria, depression, scoping review

## Abstract

**Background:**

Depression is heterogeneous and dynamic, and changes in observable behavior may accompany some of its clinical manifestations. Digital devices offer new opportunities to capture this behavior in naturalistic settings continuously. However, the specific digital features captured through device interactions and their relationships to depression-related outcomes have yet to be synthesized, and their potential relevance to functional constructs is unclear. The research domain criteria (RDoC) may provide a conceptual framework for exploring potential links between digital behaviors and functional constructs.

**Objective:**

This scoping review aimed (1) to identify and summarize key online behavioral modalities and their specific features for depression monitoring and assessment, (2) to summarize existing evidence on associations between specific behavioral features and depression-related outcomes, and (3) to explore whether the behavioral content captured by digital measures has the potential conceptual links to RDoC constructs.

**Methods:**

We conducted a scoping review of 5 databases (PubMed, Scopus, Web of Science, Embase, and PsycINFO) from inception through January 2026, supplemented by citation tracking and gray literature searches. Included studies focused on online behavioral digital phenotyping for depression and reported specific behavioral features. Two reviewers (QR and JC) independently screened studies and extracted data, with disagreements resolved through discussion or consultation with a third reviewer (YH). Findings were synthesized descriptively. Two reviewers (QR and Kaili Tang) independently assessed potential conceptual links between each behavioral modality and RDoC constructs using the official RDoC construct definitions.

**Results:**

In total, 74 studies were included. Five primary behavioral modalities were identified: call behavior (n=31), SMS (n=21), screen use (n=41), app use (n=26), and keystroke dynamics (n=14), comprising 36 core features. A total of 31 (41.9%) studies reported findings attributable to individual features, but no single feature showed a consistently directed association with depression-related outcomes across studies. An exploratory RDoC assessment suggested that candidate construct relevance primarily involved social processes, sensorimotor systems, cognitive systems, and arousal and regulatory systems.

**Conclusions:**

Online behavioral digital phenotyping captures a range of observable behaviors, but current evidence does not support interpreting individual digital behavioral features as stable markers of depression or as direct proxies for specific RDoC constructs. Future research should improve the transparency of feature definitions and reporting, strengthen the reporting of feature-level findings, and use independent validation and construct-informed study designs to enhance interpretability.

## Introduction

Depression is a major contributor to global disease burden and functional impairment [1], and timely identification and ongoing assessment are essential for effective treatment [2]. Depression is both heterogeneous and dynamic: symptom presentations vary not only between individuals but also within the same individual over time, complicating the characterization of an individual’s depressive state [3,4]. Clinical interviews, self-report measures, and clinician-administered rating scales remain central to depression assessment [5]. However, these assessments are typically conducted at discrete time points and, at least in part, rely on retrospective recall, limiting their ability to continuously capture how depression-related changes manifest in everyday life [3,6]. This limitation underscores the need for complementary measurement approaches that can continuously monitor changes in daily functioning and behavior in naturalistic settings.

In this context, digital phenotyping is defined as the continuous quantification of individual-level phenotypes using smartphones and other personal digital devices [7], offering a new way to observe everyday states and activities over time in naturalistic settings. Behavior generally refers to an individual’s observable and recordable activities [8], and some clinical manifestations of depression may be accompanied by changes in overt behavioral patterns [9]. Importantly, digital behavioral data do not directly measure depression or the underlying psychological processes. Their value lies in extending assessment into everyday life by continuously capturing changes in functioning and behavior that may not be fully represented by assessments conducted at discrete time points [3,6]. These data can be examined alongside self-reported symptoms and clinical assessments to evaluate the clinical relevance of observed behavioral changes [10]. Digital behavioral measures are therefore better positioned as complements to, rather than replacements for, conventional assessments.

Digital phenotyping encompasses multiple types of information that differ in both their sources and the content measured [11]. In this review, observable and recordable behavior in digital contexts served as the primary criterion for defining the scope of eligible digital measures. We focused on behavioral indicators generated through individuals’ interactions with digital devices. This definition emphasizes both the behavioral nature of the measure and the digital context in which the behavior occurs or is recorded, rather than encompassing all information obtainable through digital devices. For example, GPS data primarily capture location and movement in physical space, free-text data primarily provide semantic and linguistic information, and physiological sensors primarily record internal physiological states [12,13]. Although these data sources may provide complementary information relevant to depression, they fall outside the scope of digital behavior defined in this review. These device-interaction traces therefore represent a distinct class of behavioral metadata that captures how individuals interact with digital devices, rather than where they are, the semantic content of their communications, or their internal physiological states. Focusing on this class of observable behavior allowed the review to maintain a relatively consistent behavioral level of analysis.

Previous reviews have approached digital phenotyping from multiple perspectives, including broad syntheses of digital data sources and terminology [11], examinations of feature extraction and feature engineering [12,14], evaluations of passive sensing and machine learning approaches [13,15], and assessments of available smartphone datasets [16] and clinical applications [17]. Several reviews have already cataloged individual digital features or examined feature-related methodological heterogeneity [12,14,15]. However, device-interaction behaviors have generally been examined within broader digital phenotyping or passive sensing frameworks that also encompass mobility, physical activity, sleep, physiological signals, speech, and text-derived information [11-13,15]. Less attention has been paid to grouping conceptually similar device-interaction measures by the behaviors they capture and to describing variation in how these measures are operationalized across studies.

Beyond organizing these behavioral measures, further interpretation is needed to understand the psychological or functional processes that digital behaviors may reflect. The research domain criteria (RDoC), developed by the National Institute of Mental Health (NIMH) in the United States, is a transdiagnostic, dimensional research framework that organizes psychological and behavioral processes around fundamental functional constructs and examines them across multiple units of analysis, including behavior, self-report, and physiology [18-20]. Because behavior is explicitly included among the RDoC units of analysis, RDoC may provide a conceptual framework for moving from observable digital behavior to the exploratory interpretation of its potential functional significance. In this review, RDoC is not used to assign individual digital features to fixed RDoC constructs. Instead, it serves as an exploratory, construct-level interpretive framework for examining potential conceptual relationships between the behavioral content captured by digital measures and RDoC functional constructs.

Accordingly, this scoping review aimed (1) to identify and organize the specific digital behavioral features reported in the included studies and group conceptually similar measures into core features based on their behavioral measurement content; (2) to summarize the available evidence on associations between digital behaviors and depression-related outcomes, distinguishing findings attributable to individual features from those reported only at the feature-set or model level; and (3) to use RDoC as an exploratory, construct-level interpretive framework to assess the potential conceptual relevance of the behavioral content captured by digital measures to RDoC constructs.

## Methods

### Overview

This scoping review follows the framework for scoping reviews outlined by Arksey and O’Malley [21]. This study complies with the JBI Manual for Evidence Synthesis guidelines [22] and adheres to the PRISMA-ScR (PRISMA Extension for Scoping Reviews) checklist [23]. The review was registered on the Open Science Framework (registration 10.17605/OSF.IO/6X8DR). The overall scope of the review remained unchanged, with a continued focus on device-interaction behavioral digital phenotyping related to depression. During data charting and synthesis, several preregistered objectives were refined. First, to more clearly focus the research questions, the final synthesis shifted from a broader emphasis on methodological processes to a focus on digital behavioral features and the empirical evidence regarding their associations with depression-related outcomes. Second, the preregistered preliminary mapping of digital behavioral features onto RDoC was refined into an exploratory, RDoC-informed conceptual synthesis to avoid implying unvalidated one-to-one relationships between digital features and RDoC constructs. The PRISMA-ScR (Preferred Reporting Items for Systematic Reviews and Meta-Analyses Extension for Scoping Reviews) checklist is provided in Multimedia Appendix 1.

### Information Sources and Search Strategy

A systematic literature search was conducted by 2 independent researchers (YL and PZ) across 5 electronic databases: PubMed, Scopus, Web of Science, Embase, and PsycINFO. The search period spanned from each database’s inception to January 2026. Search terms were identified by consulting relevant literature and MeSH.

Our search strategy comprised three conceptual blocks: (1) depression-related terms, such as “Depression” OR “Depressive Disorder” OR “Major Depressive Disorder” OR “MDD” OR “Persistent Depressive Disorder” OR “Depressive Episode” OR “Unipolar Depression” OR “Depressive Syndrome” OR “depress”; (2) digital phenotyping terms, such as “digital biomarker” OR “digital phenotyping” OR “passive sensing” OR “smartphone” OR “wearable” OR “sensor” OR “web search” OR “text message” OR “keystroke dynamics”; and (3) detection-related terms, such as “detect” OR “screen” OR “predict*” OR “diagnosis” OR “early diagnosis.”

The search strategy was adapted to meet the specific requirements of each database. The complete search strategies are provided in Multimedia Appendix 2. Queries in each database used specific syntax and index terms. Additionally, to minimize omissions, the study used OpenSIGLE, Open Grey, the New York Medical College Library Grey Literature Report, and ClinicalTrials.gov. References and citations from included studies were also traced to supplement potentially overlooked relevant literature.

### Inclusion and Exclusion Criteria

This scoping review initially aimed to include studies focusing on online behavioral digital phenotyping for depression. Given the objective of systematically identifying key online behavioral modalities and their specific features for depression monitoring and assessment, studies that reported concrete behavioral features were prioritized for inclusion. The eligibility criteria were structured using the population, core concept, context framework (Textbox 1).

**Inclusion and exclusion criteria for this scoping review.**

**Inclusion criteria**

- Population: Individuals diagnosed with depressive disorders (eg, major depressive disorder), depressive episodes in bipolar disorder, dysthymia, or those without a formal diagnosis but assessed for depression severity using standardized scales (eg, 9-item Patient Health Questionnaire, Beck Depression Inventory, and Center for Epidemiologic Studies Depression Scale) in the general population or high-risk groups.
- Core concept: Passive collection of behavioral data via digital devices (eg, smartphones, computers, and tablets), with the data reflecting individuals’ online interaction behaviors.
- Context: Use of passively collected online behavioral data for monitoring depression-related mental health status, including symptom screening, severity assessment, or onset prediction.
- Study type: Original research.
- Language: Published in English.

**Exclusion criteria**

- Data collection: Studies that relied solely on active self-report methods (eg, ecological momentary assessment and electronic questionnaires) and did not include passive background logging.
- Study type: Nonoriginal research articles (eg, reviews, study protocols, commentaries, editorials, or case reports) or studies for which the full text was unavailable.

### Review Process

We exported citations to EndNote (Clarivate) for reference management and deduplication. Two reviewers (ML and JS) independently screened titles and abstracts of all identified studies. Studies that passed the initial screening were then assessed for full-text eligibility by 2 independent reviewers (QR and JC) using a standardized study selection form to ensure compliance with the inclusion criteria. Discrepancies were resolved through discussion or by consulting a third reviewer (YH).

### Data Extraction

We developed a standardized data extraction form based on the Joanna Briggs Institute’s scoping review methodology [24]. The form was pilot-tested on 20 studies and refined through team discussions to ensure comprehensive and consistent data collection. For each included study, 2 researchers (QR and JC) independently extracted data using a standardized Microsoft Excel–based form. Any discrepancies during the extraction process were resolved through discussion or by consulting a third researcher (YH) to reach consensus.

Consistent with the objectives of this review, the extracted data primarily covered three areas: (1) study characteristics, including first author, publication year, country or region, study design (eg, cross-sectional or longitudinal cohort), and study duration; (2) participant characteristics, including sample size, target population (clinically diagnosed patients vs general or high-risk populations), and instruments used to assess depression; and (3) data modalities, including types of digital behavioral data (eg, calls, SMS, screen use, app use, and keystroke dynamics) and the specific behavioral features reported in the original studies.

After the core features of each modality were identified, 2 reviewers (QR and Kaili Tang), one with a psychology background, independently assessed whether the behavioral measurement content captured by each modality had a potential conceptual link to any of the 25 RDoC constructs, using the official NIMH RDoC construct definitions as a reference [25]. Each of the 5 behavioral modalities was evaluated against all 25 RDoC constructs, yielding 125 modality-construct judgment units. Judgments were based on the behavioral content actually measured by each modality and the corresponding official RDoC construct definitions. Initial exact agreement and Cohen κ were calculated before consensus discussion [26]; disagreements were then discussed until final consensus was reached.

## Results

### Overview

We identified 25,746 records through database searches, reference and citation tracking, and gray literature searches. After removing duplicates, 9863 records identified through database searches were screened by title and abstract, and 9661 were excluded. An additional record was identified through citation tracking. The full texts of 203 reports were assessed for eligibility. Ultimately, 129 reports were excluded, and 74 studies were included in this review. Figure 1 shows the process of study identification and screening.

**Figure 1:**
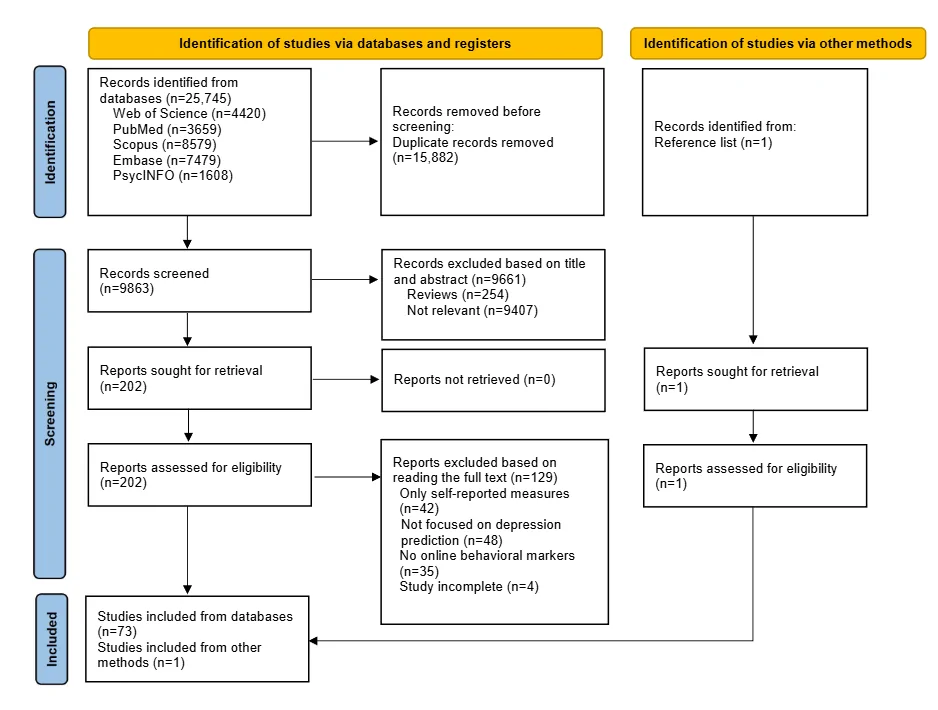
PRISMA (Preferred Reporting Items for Systematic Reviews and Meta-Analyses) flow diagram of the literature selection.

### General Information About the Included Studies

This scoping review included 74 studies [27-100] that used online behavioral data collected from mobile devices to identify markers of depression. Five categories of online behavioral modalities were identified across these studies, with the following frequencies: call behavior (n=31), screen use (n=41), app use (n=26), SMS (n=21), and keystroke dynamics (n=14). Figure 2 shows the frequency distribution of studies examining each behavioral modality, and Figure 3 illustrates the number of studies using different combinations of these modalities.

**Figure 2:**
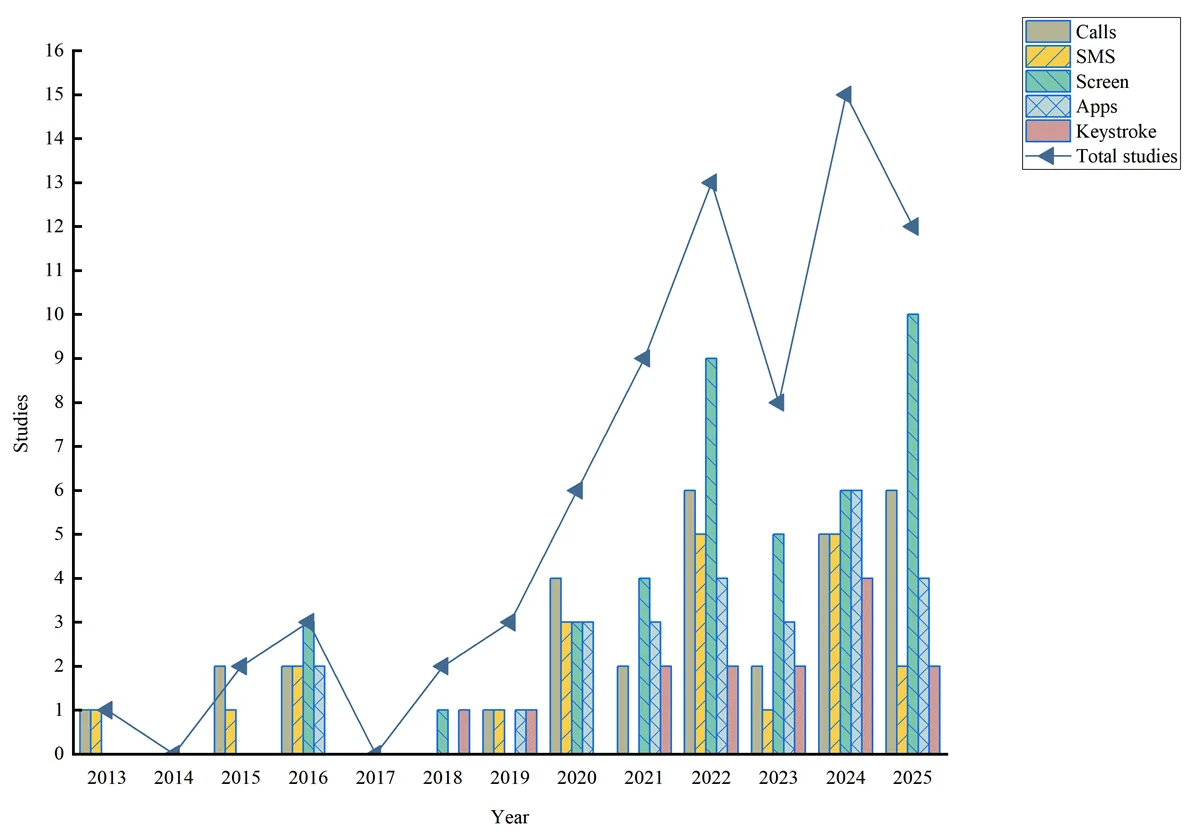
Temporal trend of included studies by behavioral modality.

**Figure 3:**
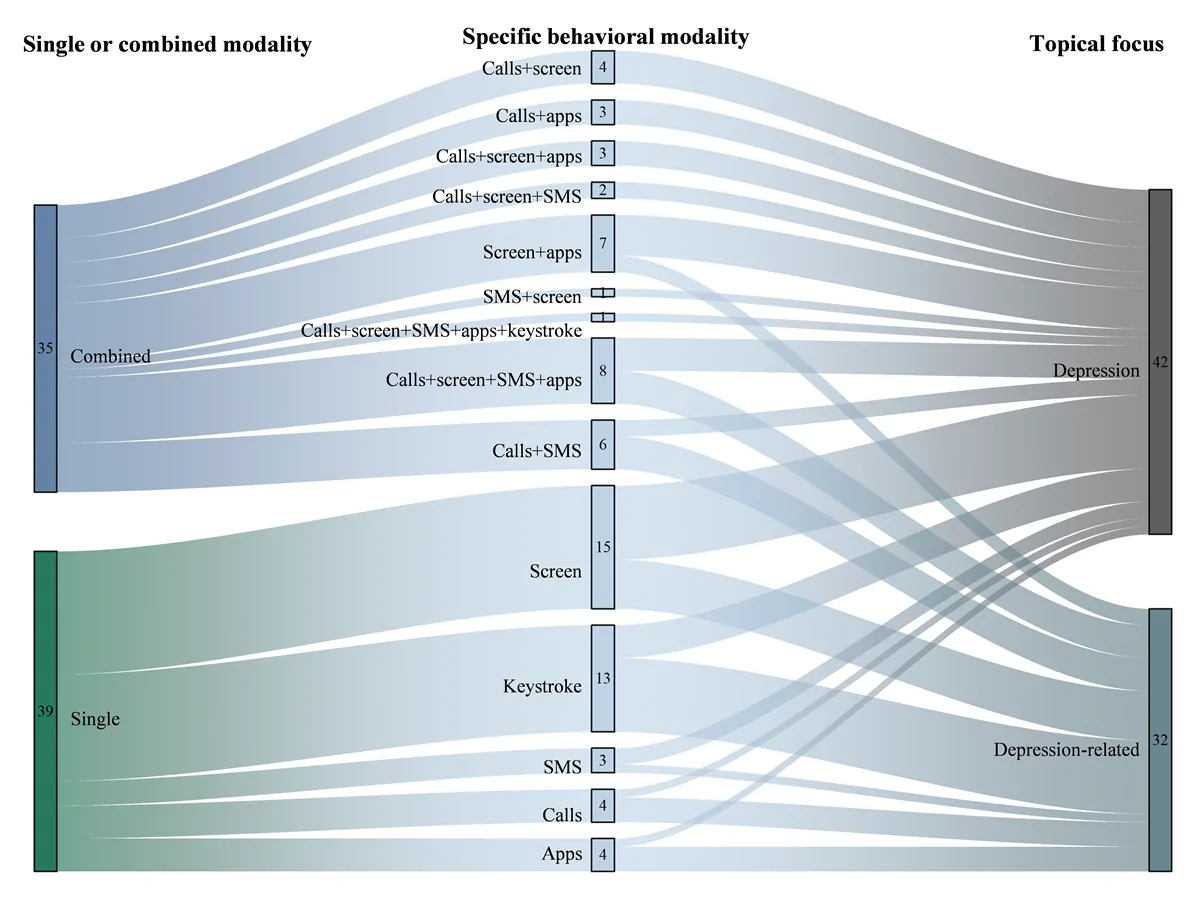
Sankey diagram of included studies. Studies were categorized by modality use and topical focus. Single-modality studies analyzed 1 of the 5 modalities included in this review, whereas combined-modality studies analyzed 2 or more. This classification was based solely on these 5 modalities. Depression-focused studies primarily examined depression, depressive symptoms, or depressive states. In contrast, depression-related studies primarily focused on mood, mental health, or other psychiatric or functional outcomes rather than depression itself.

Of the 74 studies, 56 were longitudinal, and 18 were cross-sectional. Studies were classified as longitudinal if they involved repeated or temporally ordered observations of the same participants over a follow-up period; those without temporal follow-up were classified as cross-sectional. The majority of the studies were published between 2020 and 2025. A subset of 24 studies included participants with clinically diagnosed psychiatric disorders. Where reported, diagnoses were based on the *Diagnostic and Statistical Manual of Mental Disorders, Fifth Edition* (DSM-5) [27-36], *International Classification of Diseases, 10th Revision* (ICD-10) [37-39], or *Diagnostic and Statistical Manual of Mental Disorders, Fourth Edition, Text Revision* (DSM-IV-TR) criteria [40], using diagnostic instruments such as the Mini-International Neuropsychiatric Interview [30-36,41], Structured Clinical Interview for DSM Disorders [34-36,42-44], or Schedules for Clinical Assessment in Neuropsychiatry [39]. For the subjective assessment of depressive symptoms, the 9-item Patient Health Questionnaire (PHQ-9; n=31) was the most commonly used self-report instrument. Objective behavioral data were primarily collected through dedicated smartphone apps or monitoring platforms; 40 studies were Android-only, 5 iOS-only, 10 supported both systems, and 19 did not report operating system compatibility. Detailed characteristics of the included studies are provided in Multimedia Appendix 3 [27-100]. Among the 74 included studies, 22 conducted secondary analyses of existing research datasets [30,39,43,45-63]. Datasets with identifiable access pathways or explicit statements of public availability are summarized in Table 1.

**Table 1:** Reusable datasets with identifiable access pathways among the included studies.

Dataset	Data relevant to this review	Access status	Access route	Associated studies
StudentLife	Passive smartphone sensing and mental health assessments	Directly accessible	StudentLife public repository	[45,48-50,57]
DepreST-CAT	Call and SMS logs with PHQ-9^a^ and GAD-7^b^ assessments	Directly accessible for academic use	GitHub repository	[46]
DemonicSalmon	Call and SMS logs with mental health assessments	Reported as publicly available; portions currently accessible	OSF^c^	[47]
BRIGHTEN	Passive smartphone sensing and clinical assessments	Controlled access	Synapse	[58]
GLOBEM	Longitudinal smartphone sensing and mental health assessments	Controlled access	PhysioNet	[52]

^a^PHQ-9: 9-item Patient Health Questionnaire.

^b^GAD-7: 7-item Generalized Anxiety Disorder.

^c^OSF: Open Science Framework.

### Landscape and Reporting of Online Behavioral Features

The digital behavioral features reported across the included studies were grouped into 5 modalities: calls, SMS, screen use, app use, and keystroke dynamics, comprising 36 core features in total (Table 2). Conceptually similar measures were often described with different terms across studies. For example, the interval between consecutive keystrokes was variously described as the interkey interval, interkey delay, or flight time [40,64,65]. In contrast, the interval between pressing and releasing a key was described as the hold time, dwell time, or keypress duration [53,54,65]. To facilitate cross-study comparison, we retained the terminology used in the original studies and grouped conceptually similar measures into harmonized core features.

The operationalization of the core feature also varied across studies. Differences were observed in behavioral direction or status, contextual or stratification scope, timing, aggregation window, and statistical summary. For example, call behavior was differentiated by incoming, outgoing, answered, or missed status [38]; screen use was defined by device states such as screen-on or unlocked or by interaction units such as sessions [66,67]; and keystroke timing features were summarized using means, medians, percentiles, and measures of variability [56,68]. The level of operational detail also varied, ranging from broad feature labels such as “call count” [69] to more detailed specifications of behavioral context, timing, aggregation windows, and statistical summaries [38,41,56,68]. Table 2 summarizes the core features and the corresponding operational dimensions and variants explicitly reported in the included studies. The complete set of original feature names, harmonized core features, operational characteristics, and study-level sources is provided in Multimedia Appendix 4.

**Table 2:** Core smartphone-derived behavioral features and reported operational dimensions^a^.

Modality and core feature			Operational dimensions and reported variants
**Calls**			
	Call count	Context: incoming/outgoing; answered/not reached; all contacts, phonebook contacts, top-5/top-10 contacts. Timing: morning, afternoon, evening, nonworking hours. Time frame: hourly, daily, previous 24 hours, 3/7/14/30-day rolling window. Summary measures: total, mean, proportion, entropy/normalized entropy, maximum spectral frequency; mean daily call frequency, frequency.	
	Call duration	Context: incoming/outgoing; missed/connected; all contacts, phonebook contacts, top-5/top-10 contacts. Timing: morning, afternoon, evening, nonworking hours. Time frame: daily, 3/7/14/30-day rolling window. Summary measures: total, mean, median, SD, entropy/normalized entropy, wavelet entropy, spectral roll-off.	
	Missed-call count and rate	Context: missed, unanswered, unreturned, incoming. Time frame: daily. Summary measures: count, fraction.	
	Distinct call contacts	Context: incoming, outgoing, missed; call contacts and saved contacts. Time frame: daily.	
	Call direction and status balance	Context: outgoing versus incoming; outgoing among connected calls. Summary measures: fraction, ratio, proportion.	
**SMS**			
	SMS count	Context: incoming/received, outgoing/sent; top-5/top-10 contacts. Timing: morning, afternoon, evening. Time frame: daily, 2-week. Summary measures: total, mean, proportion, entropy/normalized entropy, wavelet entropy, spectral spread/roll-off, maximum spectral frequency.	
	SMS message length	Context: incoming, outgoing; top-10 contacts. Timing: morning, afternoon, evening. Time frame: daily. Summary measures: total, mean, SD, entropy/normalized entropy.	
	SMS reply count	Context: incoming-to-outgoing reply sequence. Time frame: 2 weeks. Summary measures: count.	
	SMS reply latency	Context: incoming-to-outgoing reply sequence. Time frame: 2 weeks. Summary measures: minimum, median/P50, maximum, P10, P25, P75, P90, boxplot outlier count.	
	Distinct SMS contacts	Context: incoming, outgoing, overall; contacts responded to. Time frame: 2 weeks.	
	SMS direction and reciprocity balance	Summary measures: sent-message ratio; P10, P25, median/P50, P75, P90, boxplot outlier count.	
	SMS conversation continuity and bursts	Summary measures: consecutive-message count; P10, P25, median (P50), P75, P90, boxplot outlier count.	
**Screen**			
	Screen unlock and state-event count	Context: unlock, screen-on/display-on, screen-off, phone open, lock/unlock; overall, dormitory, study places. Timing: evening; 0-6 AM, 6 AM-12 PM, 12-6 PM, 6 PM-12 AM. Time frame: daily, 2 weeks. Summary measures: count, frequency/rate, mean, SD.	
	Screen-use duration	Context: screen-on, unlocked, online session, general phone/screen use; overall, dormitory, study places. Timing: 0-6 AM; 6 AM-12 PM; 12-6 PM; 6 PM-12 AM; daytime/nighttime, evening; four 6-hour periods. Time frame: episode/session, daily, weekly, 2 weeks, hourly. Summary measures: total, mean, median/quartiles, minimum/maximum, SD, proportion.	
	Screen inactivity and screen-off duration	Context: screen lock, phone inactive, dark screen, off period; dark-screen interval >1 hour. Time frame: daily.	
	Screen session count and structure	Context: phone session, online session, screen-use episode. Timing: morning, afternoon, night, midnight. Time frame: hourly, daily, fortnightly. Summary measures: count, mean session duration.	
	Screen-use timing and regularity	Timing: screen-off timestamp, first unlock, first use after midnight; four 6-hour periods; daytime/nighttime; weekdays/weekends/all days. Context: smartphone activity during >5% of the sleep period; hourly screen-on percentage >10%. Time frame: daily, 2 weeks. Summary measures: number of days with phone use, regularity index, circadian rhythm, entropy/normalized entropy, and inter-unlock interval.	
	Screen-derived sleep-wake timing	Context: phone inactivity. Time frame: daily. Summary measures: minimum, Q1, median (Q2), mean, Q3, maximum, SD.	
	Screen touch interaction	Context: tap/click, long tap/long click, scroll. Summary measures: count, intertouch interval.	
**Apps**			
	App-use count	Context: use; launch; open/close; foreground episode; crash; significant apps; nonsignificant apps; app categories; per-app; top-5 apps; communication; social media; leisure; game; Weibo/social apps. Timing: morning, afternoon, evening, all day. Time frame: previous 60 minutes, daily. Summary measures: count; event frequency; SD.	
	App-use duration	Context: app categories; significant/nonsignificant apps; per-app/top-5 apps; foreground apps/activity. Time frame: previous 60 minutes, daily, weekly; app-use episode. Summary measures: total, mean, daily average.	
	App repertoire and diversity	Context: foreground apps, launched apps, significant/nonsignificant apps. Timing: 00:00-05:00. Time frame: daily. Summary measures: distinct/unique app count, entropy/normalized entropy, 24-hour per-app frequency-distribution entropy.	
	App switch count	Time frame: daily. Summary measures: switch count.	
	App-use timing and regularity	Timing: first app use, last app use; daytime versus nighttime. Summary measures: app-use regularity index, daytime-to-nighttime post ratio.	
	App and notification response	Context: notification categories; click, acceptance, handled on another device; seen, decision, response stages. Summary measures: count, rate, mean response/interaction time.	
	App content feature	Context: Twitter/Instagram; hashtags, mentions, attached media, captions/posts; positive/negative content; location/geotag/source; image hue/brightness/saturation; likes/comments/favorites; daily photos. Time frame: daily. Summary measures: count, mean.	
	App use (general app-use indicators with unspecified underlying quantity)	Context: email, browser, game, social media, app-based messaging; Twitter active actions; top-5 apps. Time frame: daily. Summary measures: active-action ratio, summed use.	
**Keystroke**			
	Interkey interval	Context: interkey/flight, press-to-press, release-to-release; press-to-flight ratio, movement speed. Time frame: session, weekly. Summary measures: mean, median, quantiles/P95, MAD^b^, RMSSD^c^, mean absolute deviation, mean change.	
	Keystroke dwell and hold time	Context: hold time, dwell time, keypress duration. Time frame: session, weekly. Summary measures: median, quantiles, variability, mean change, mean.	
	Typing speed	Summary measures: typing speed, characters per minute.	
	Typing pause	Summary measures: pause occurrence, pause ratio.	
	Typing volume and session structure	Context: keystroke/keypress count, characters; session count/length/duration; keypresses/characters per session; null/space/nonalphanumeric keys; active/upright sessions; typing bursts. Time frame: daily, weekly. Summary measures: count, proportion/rate.	
	Typing rhythm and regularity	Timing: diurnal typing-session distribution. Summary measures: typing rhythm, circadian keypress-pattern similarity.	
	Typing error and correction	Context: delete, autocorrect; typing error; Euclidean/Manhattan key-error distance. Time frame: session, daily. Summary measures: rate, distance.	
	Backspace rate and count	Time frame: session, daily, weekly. Summary measures: count, ratio, rate, proportion, weekly SD of daily backspace rate.	
	Keystroke pressure	Operationalization details: not further specified.	

^a^Operational dimensions were summarized descriptively from qualifiers explicitly reported in the included studies. No additional assumptions were made when temporal aggregation or computational procedures were not clearly specified.

^b^MAD: median absolute deviation.

^c^RMSSD: root mean square of successive differences.

### Depression-Related Findings Across Behavioral Modalities

#### Overview

As summarized in Table 3, we categorized depression-related findings into individual feature-level results, single-modality feature-set or model results, and multimodal results. Feature-level findings included associations and between-group or between-state differences attributable to individual behavioral features. Findings reported only at the single-modality feature-set/model level or at the multimodal level were not attributed to specific features unless the original study reported feature-specific effects.

**Table 3:** Levels and modality composition of depression-related results across included studiesa.

Result category and modality or combination		Studies (N=74), n (%)
Individual feature-level results		
	Any feature-level result	31 (41.9)
	Calls	11 (14.9)
	SMS	8 (10.8)
	Screen	17 (23)
	Apps	10 (13.5)
	Keystroke	9 (12.2)
**Single-modality feature-set or model results**		
	Any single-modality result	13 (17.6)
	Calls	2 (2.7)
	SMS	4 (5.4)
	Screen	2 (2.7)
	Apps	0 (0)
	Keystroke	5 (6.8)
**Multimodal results**		
	Any multimodal result	52 (70.3)

^a^Categories were not mutually exclusive, as individual studies could report results at multiple levels and across multiple modalities. Percentages were calculated using all 74 included studies as the denominator.

#### Calls

Findings for call frequency were mixed. One study found that fewer answered incoming calls were associated with higher Hamilton Depression Rating Scale scores; in the same study, answered incoming and outgoing call counts were lower during depressive than euthymic states [38]. Two additional studies reported lower call activity/fewer incoming or outgoing calls among participants with depression than among those without depression [70,71]. In contrast, other studies found no significant association or group difference in call count or frequency across continuous symptom scores, depression groups, or patient-control comparisons [28,34,36,45,58].

Findings on call duration also varied in direction. Some studies found that longer incoming or outgoing call duration was associated with higher depression scores [39,72]. In contrast, others reported negative associations with symptom severity or shorter call duration among participants with depression [27,34,71]. In another study, incoming call duration was negatively associated with PHQ-9 scores, whereas outgoing call duration was positively associated [36].

Other call-related measures were examined less frequently. The proportion of missed incoming calls was positively associated with depression severity and was higher during depressive than euthymic states in one study [38]. Another study found that unsuccessful outgoing calls differed significantly from euthymic periods only during the late pre-episode phase, with no significant differences in the other depressive-episode or pre-episode comparisons [43]. At the single-modality feature-set/model level, calls-only models achieved an accuracy of 64.2% in one study and *F*_1_-scores ranging from 0.71 to 0.78 in another [28,37].

#### SMS

Findings for SMS volume were also inconsistent. One study found that participants with depression sent more SMS messages than those without depression [71], whereas another reported a negative association between SMS frequency and depression severity [27]. Other studies found no significant association or group difference in SMS volume across momentary depressed mood, clinical depression status, or patient-control comparisons [28,34,36,58].

Other SMS features, including message length, contact count, reply latency, sent-received reciprocity, and consecutive-message patterns, were primarily examined within multifeature predictive analyses, with relatively few findings attributable to individual features [27,28,38,46,47,58,63,64,69]. At the single-modality feature-set/model level, internal validation performance varied across SMS feature sets, with balanced accuracy ranging from approximately 0.59 to 0.81 [46,47,63]; an SMS-only model in another study achieved an *F*_1_-score of 0.80 [28].

#### Screen

Findings on overall screen use duration and frequency were mixed. Several studies found that longer screen-on, unlocked, or overall screen-use duration was associated with higher 8-item Patient Health Questionnaire (PHQ-8) or PHQ-9 scores or a greater likelihood of depression [41,49,72,73]. One study examining broader mental health outcomes found that greater overall screen time was associated with higher internalizing symptoms, whereas unlock count was inversely associated [74]. In contrast, other studies found no significant association or group difference between overall screen-use duration or frequency and depression-related outcomes [27,34,36,67,75,76].

Screen-use timing showed additional variation. One study found that participants with depression first used their phones earlier after midnight than those without depression [60]. Daytime use duration was negatively associated with PHQ-9 scores in 2 studies, whereas findings for nighttime use duration differed in direction across studies [29,77].

In one of these studies, several associations were observed among iOS users, whereas most corresponding measures were nonsignificant among Android users [29].

In an episode-monitoring study, screen display duration differed between depressive and euthymic periods in only 1 comparison, and no screen measures differed significantly in the pre-episode comparisons [43]. At the single-modality feature set/model level, sleep features derived from screen lock and unlock events achieved an area under the receiver operating characteristic curve of 0.82 in one study. In contrast, a screen-derived family of sleep features achieved a classification accuracy of 55.56% in another [48,66].

#### Apps

One study found that evening social app use was more frequent among participants with depression than among those without. The same pattern was observed among male participants, whereas female participants with depression showed more frequent morning Weibo use [70]. Another study reported differences in several app-opening and use-intensity measures across levels of depressive symptom severity [78].

Associations with specific app categories also differed by operating system. Among iOS users, social app use duration and gaming and shopping app session counts were positively associated with PHQ-9 scores, whereas email app use duration was negatively associated with PHQ-9 scores. Among Android users, video and gaming app use durations were positively associated with PHQ-9 scores, whereas shopping app use duration was negatively associated [29]. Other studies found no significant associations between most app measures and PHQ-8 or PHQ-9 scores, depression status, or symptom change [34,36,51,57].

Findings also varied by observation window and analytical level. App-use and notification measures calculated over the preceding 14 days were more strongly associated with current PHQ-8 scores than measures calculated over 7 days or on the assessment day alone [79].

#### Keystroke Dynamics

Findings for keystroke timing differed across measures and symptom dimensions. One study found a positive association between interkey delay and Hamilton Depression Rating Scale scores [40]. Another reported a positive association between median key hold time and PHQ-9 scores [65]. In another study, a weak negative association between psychological distress and keystroke latency remained significant after correction for multiple comparisons (*r*=–0.07) [54]. Longer interkey intervals were also associated with greater anhedonia in one study, whereas shorter interkey intervals or greater keypress activity were associated with greater irritability or social dysfunction; these findings were based on uncorrected *P* values [56].

Backspace-related findings also depended on how the measure was operationalized. One study found no significant difference in mean backspace rate between participants with mood disorders and healthy controls, although depression scores differed across derived backspace phenotypes [31]. At the single-modality feature-set/model level, 3 studies reported areas under the receiver operating characteristic curve ranging from 0.89 to 0.98 for models combining multiple keystroke features [53,55,65].

#### Multimodal Results

Multimodal results were reported in 52 (70.3%) studies. Because these analyses combined features from multiple behavioral modalities, their overall findings were not attributed to any single modality or individual feature unless feature-specific effects were reported. For example, models combining aggregate phone-use and app-category features achieved *F*_1_-scores ranging from 0.71 to 0.80, whereas another multimodal behavioral model achieved an *F*_1_-score of 0.87 [29,78]. Person-specific multivariate analyses combining app and screen measures identified significant relationships in 4 of 5 participants, although the contributing measures and their directions differed across individuals [80]. Models that additionally incorporated data modalities outside the scope of this review were not further synthesized at the modality level.

### Exploratory RDoC-Informed Conceptual Framework

Beyond their empirical associations with depression-related outcomes, we further explored the potential conceptual links between the behavioral content captured by these digital features and RDoC constructs. Before the consensus discussion, the 2 reviewers agreed on 117 of 125 (93.6%) modality-construct judgments, yielding a Cohen κ of 0.697. Disagreements were resolved through discussion to establish the final candidate construct relevance and the interpretive boundaries presented in Table 4. To provide a visual overview of this exploratory synthesis, Figure 4 presents the relationships among the candidate RDoC domains identified earlier, the 5 behavioral modalities, and representative digital features, and includes selected depression-related clinical manifestations as examples of clinical context. These elements are intended to aid interpretation and do not imply direct correspondences between clinical manifestations and RDoC constructs.

**Table 4:** Exploratory research domain criteria (RDoC) construct relevance of behavioral content captured across digital modalities^a^.

Modality	Behavioral measurement content (core features)	Candidate RDoC domain and construct	Interpretive boundaries
Calls	Call count; call duration; missed-call count and rate; distinct call contacts; call direction and status balance	Social processes: affiliation and attachment; social communication	Communication metadata do not directly capture relationship quality, emotional attachment, or subjective social experience.
SMS	SMS count; SMS message length; SMS reply count; SMS reply latency; distinct SMS contacts; SMS direction and reciprocity balance; SMS conversation continuity and bursts	Social processes: affiliation and attachment; social communication	SMS metadata do not directly capture the semantic or emotional content of communication, relationship quality, or subjective affiliation.
Screen	Screen unlock and state-event count; screen-use duration; screen inactivity and screen-off duration; screen session count and structure; screen-use timing and regularity; screen-derived sleep-wake timing; screen touch interaction	Arousal and regulatory systems: circadian rhythms; sleep-wakefulness Sensorimotor systems: motor actions	Screen inactivity is not equivalent to sleep, device-use timing does not directly measure circadian rhythms, and touchscreen interactions provide only indirect information about motor actions.
Apps	App-use count; app-use duration; app repertoire and diversity; app switch count; app-use timing and regularity; app and notification response; app content feature; app use	Social processes: social communication Arousal and regulatory systems: circadian rhythms	Interpretation depends on app category and use context; app use does not necessarily indicate social communication or endogenous circadian rhythms.
Keystroke	Interkey interval; keystroke dwell and hold time; typing speed; typing pause; typing volume and session structure; typing rhythm and regularity; typing error and correction; backspace rate and count; keystroke pressure	Cognitive systems: attention; cognitive control Sensorimotor systems: motor actions	Keystroke dynamics are influenced by device, task, and context and are not specific measures of cognitive or motor constructs.

^a^Candidate links were judged based on the behavioral content actually captured by each modality, with reference to the official NIMH RDoC construct definitions. These links do not indicate that established or validated correspondences between digital behavioral features and RDoC constructs have been demonstrated.

**Figure 4:**
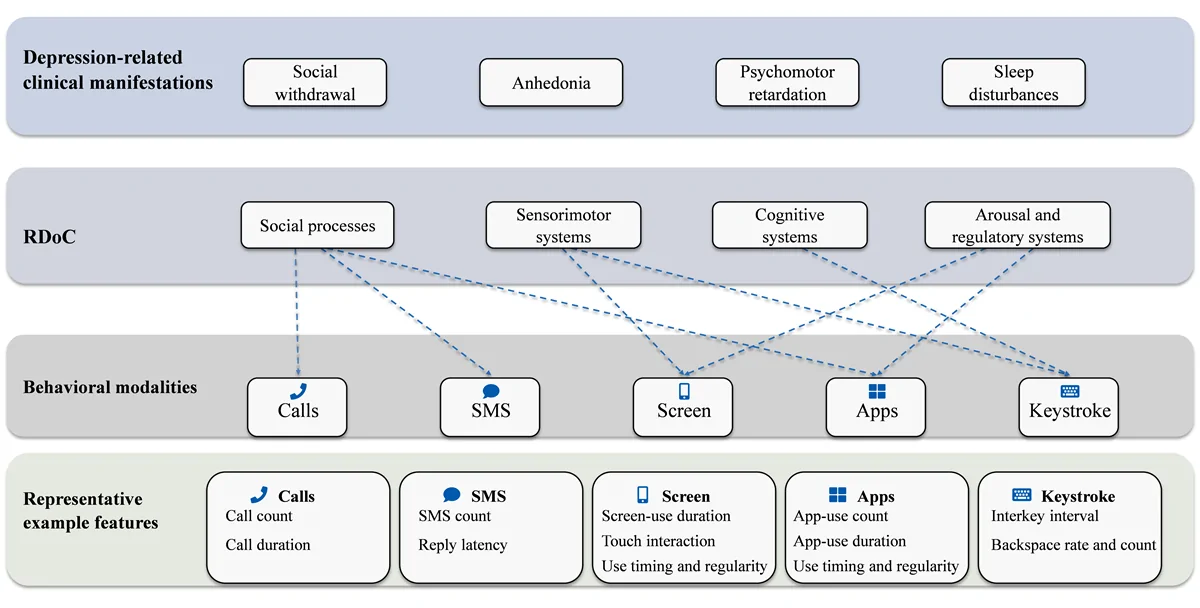
Conceptual framework for exploring digital behavioral features in depression from an RDoC-informed perspective. Representative features were selected from commonly reported behavioral measures to illustrate the primary measurement content of each modality. RDoC: research domain criteria.

## Discussion

### Principal Findings

This review found that the literature covered a broad range of digital behavioral features; yet, the specific features within each modality remained fragmented across studies. Even when studies examined conceptually similar behaviors, they differed substantially in terminology, definitions, and operationalization. Associations between digital behavioral features and depression-related outcomes were also highly heterogeneous. An exploratory analysis using the RDoC framework further suggested a potentially many-to-many relationship between digital behaviors and psychological constructs: the behavioral content captured by a modality may be relevant to multiple constructs, while the same construct may manifest through different digital behaviors. The available evidence does not support a stable correspondence between specific digital features and RDoC constructs.

### Heterogeneity in Feature Definitions and Operationalization

Feature-level heterogeneity arose not only from the selection of different behavioral indicators across studies but also from variations in the terminology, definitions, and operationalization of conceptually similar measures. Even when indicators were grouped under the same core feature, they could capture different behavioral content depending on behavioral direction or state, temporal constraints, context, aggregation windows, and statistical summaries. For example, across the included studies, screen-use duration was operationalized using different definitions, including screen-on time, unlocked time, and general phone-use duration. It was summarized across different temporal scales [41,60,66,81]. Comparisons based solely on feature names may therefore obscure important differences in what was actually measured. Previous reviews have likewise reported substantial variation in feature construction and methodological reporting [14,101], which may limit cross-study comparison and reproducibility.

In this review, indicators were grouped by modality and core feature to organize and summarize the literature; this grouping does not imply that indicators within the same category are measurement-equivalent or interchangeable. Differences in operationalization should also not necessarily be regarded as methodological shortcomings, as they may reflect different research questions, observational scales, and analytical objectives. Rather than requiring all studies to adopt uniform feature definitions, a more practical priority is to clearly report the behavioral content represented by each feature, its specific definition, and relevant temporal, contextual, aggregation, and statistical specifications to improve interpretability and comparability. Methodological work in digital phenotyping has similarly emphasized that decisions made during data collection, processing, and analysis can influence the interpretation and reproducibility of study findings [102].

Such transparency also involves balancing data sharing with participant privacy. Passively collected smartphone data may contain sensitive and highly individualized information; therefore, improving research transparency does not necessarily require unrestricted sharing of individual-level raw data [103,104]. In this review, 61 of the 74 (82%) included studies reported at least 1 specific privacy-protection measure (eg, only sensor metadata are extracted; all numbers are processed with one-way hashing) [37,82]. For sensitive data, controlled or managed access may be considered in accordance with data-governance requirements and the scope of informed consent, while sharing appropriate analytical code may improve transparency and support independent reproducibility [102,104].

### Consistency and Heterogeneity in Depression-Related Findings

Among studies in which results could be attributed to individual features, evidence for reproducible and directionally consistent associations between specific behavioral indicators and depression-related outcomes was limited. For call behavior, several studies found that greater depressive symptom severity or depressive states were associated with lower call activity [38,70,71], whereas others reported no significant associations [28,34-36,45,58]. Screen-use findings were similarly inconsistent. Longer overall screen-use or unlock duration was associated with higher depression levels in some studies [41,49,72,73], whereas others reported negative or nonsignificant associations [27,34,36,67,76,77]. Depression-related differences were also reported for more specific temporal and interaction-direction measures, including nighttime use, first use after midnight, and incoming versus outgoing calls. Still, these findings likewise did not converge in direction [27,29,36,38,60,77]. Thus, characterizing the relationship between digital behavior and depression solely in terms of increases or decreases in overall activity may be overly simplistic, as depression-related differences have also been observed in more specific dimensions such as behavioral timing and interaction direction.

This heterogeneity should not necessarily be interpreted as contradictory relationships between the same digital behavior and depression, because studies differed in the specific behaviors measured, the effects estimated, and the analytical conditions under which those effects were examined. Some of these differences were evident even within the same study. For example, call frequency and duration did not differ significantly between participants with major depression and healthy controls. However, longitudinal within-person analyses using person-mean-centered predictors showed that, relative to each participant’s own average behavior, shorter incoming call duration (β=–.08) and longer outgoing call duration (β=.05) were associated with higher PHQ-9 scores [36]. This illustrates that between-group differences and within-person longitudinal associations represent effects at different levels and should not be interpreted equivalently. Similarly, the depressed group received fewer calls in the group comparison, but call-related measures did not remain significant in the subsequent multivariable analysis [70]. Screen behavior also varied substantially in operationalization, with studies using measures such as unlock duration, screen-on duration, overall phone-use duration, online duration, and use within specific times of day [27,29,41,60,66,77]. Associations of several screen- and app-use measures with PHQ-9 scores also differed between iOS and Android users in one of the included studies [29]. Cross-study heterogeneity, therefore, needs to be interpreted in light of differences in feature definitions, depression-related outcomes, levels of analysis, statistical procedures, and technological platforms. In addition, the nonsemantic digital behavioral measures included in this review primarily capture the frequency, duration, and timing of behavior and generally provide limited information about its purpose, context, or subjective experience. The observed cross-study differences therefore cannot currently be attributed to any single behavioral or psychological mechanism.

Similar interpretive limitations apply to multifeature prediction models. Although combinations of digital behavioral features may distinguish or predict depression, model-level performance does not establish the independent contribution or real-world behavioral meaning of individual features. Future studies could therefore complement overall predictive performance with estimates of feature- or feature-group contributions, where appropriate, and use independent behavioral, self-report, or functional measures to evaluate the processes that these digital behaviors may reflect.

### From Digital Behavioral Features to Construct-Level Interpretation: An Exploratory RDoC Perspective

Digital behavioral indicators do not inherently carry fixed psychological meanings. The measures included in this review primarily characterize device-interaction behavior in terms of frequency, duration, timing, direction, state, regularity, and dynamic variation. Interpreting these digital traces in relation to real-world functional processes or theoretical constructs therefore requires additional inference and validation [13]. Associations between digital features and depression-related outcomes may indicate clinical relevance, but they do not establish which specific functional processes those features reflect. RDoC organizes functional constructs across multiple units of analysis, including behavior, self-report, and physiology [18-20], thereby providing a theoretical framework for examining potential links between digital behavior and these constructs. Previous work has also suggested that smartphones and other connected devices can extend the measurement of behavior in naturalistic and longitudinal settings within an RDoC framework [105]. Accordingly, rather than assigning individual digital indicators to fixed RDoC constructs, we identified candidate links based on the behavioral content captured by each modality. Call and SMS data primarily reflect communication frequency, direction, and contact patterns; screen data capture duration and temporal patterns of device use as well as some touchscreen interactions; app data reflect the frequency, duration, categories, and timing of app use; and keystroke data capture fine-grained interaction characteristics such as typing speed, pauses, and corrections.

Related empirical research provides preliminary support for some links between digital behavior and real-world functional processes, with social interaction representing one of the clearer examples. Contact-network size, communication frequency, call duration, and reciprocity derived from call and SMS data have been associated with self-reported diversity of social roles and frequency of social contact, suggesting that communication metadata can capture observable aspects of social integration and interaction [106].

Longitudinal studies further suggest that depression-related social changes involve not only network structure but also relationship intimacy, support, and conflict [107,108], dimensions that cannot be determined from communication metadata alone. Call and SMS metadata may therefore provide behavioral clues relevant to the social processes domain. Still, their links to specific RDoC constructs, such as affiliation and attachment or social communication, require validation with independent measures.

Other modalities also provide empirical clues regarding the potential functional significance of digital behavior. Temporal patterns of smartphone interactions, particularly screen and app use, may provide indirect clues about sleep-wake behavior. Sleep-wake timing estimated from smartphone touchscreen interaction timestamps has shown substantial correspondence with wrist-actigraphy estimates [109]. Additionally, screen- and app-use timing has been used to characterize sleep-wake and circadian activity patterns [110]. Although calls and SMS also contain temporal information, their timestamps are typically tied to discrete communication events, whereas screen and app measures capture broader patterns of interaction timing and duration across the day [38,46,60,66]. Screen and app timing may therefore provide relatively more direct behavioral clues about sleep-wake and daily activity rhythms, while temporal features from calls and SMS may offer complementary information. Fine-grained touchscreen dynamics may also provide information relevant to motor interaction, although their relevance to motor actions depends on task and context. Naturalistic smartphone keyboard studies suggest that keystroke dynamics, including typing speed and interkey intervals, may reflect aspects of processing speed and executive functioning [83]. A systematic review and meta-analysis further indicated that keystroke-based measures may provide information relevant to fine motor function [111]. In addition, studies that use keystroke patterns to construct brain-age indicators suggest that these features may contain information about broader individual functional variation [68]. These findings suggest that keystroke dynamics may capture information relevant to both cognitive and sensorimotor functioning. However, their links to specific RDoC constructs such as cognitive control or motor actions remain to be established. The functional interpretation of app-use data is even more dependent on app category, purpose of use, and context. By comparison, the nonsemantic device-interaction and communication metadata included in this review are less suited to directly capturing processes related to threat and loss within the negative valence systems or to reward anticipation, reward learning, and reward valuation within the positive valence systems [25]. The uneven observability of RDoC domains in this review therefore appears to reflect the behavioral scope of the included data modalities rather than differences in the importance of these domains to digital phenotyping for depression.

Future research could further explore multilevel relationships among digital behaviors, real-world functional processes, and RDoC constructs. A construct-driven approach could begin by defining a target clinical or functional construct, hypothesizing its potential behavioral manifestations in everyday life, and selecting digital behavioral modalities and specific measurement features that are theoretically aligned with these behavioral manifestations [105,112]. Subsequently, these digital measures could be evaluated alongside independent assessment approaches, including ecological momentary assessment, self-report, cognitive or behavioral tasks, and physiological measures, to examine whether they validly reflect the intended constructs [112]. Consistent with the V3 framework, digital measures should be evaluated through verification, analytical validation, and clinical validation, depending on their intended use [113]. Accordingly, the RDoC analysis in this review is best regarded as an exploratory, construct-level interpretive framework for identifying potential links between digital behavior and functional constructs, rather than for establishing validated mappings.

### Limitations

This review has several limitations. The terminology and level of detail used to report behavioral features varied considerably across studies. Although we organized and categorized features as closely as possible based on information explicitly reported in the original studies, some interpretive bias may remain. In addition, this review focused only on device-interaction and communication metadata. It did not include other digital phenotyping data sources, such as location and mobility data, physiological signals, free-text content, or actively collected self-reports. The synthesized features therefore capture only part of the behavioral and functional information available through digital phenotyping. Some studies also reused the same existing datasets, potentially overrepresenting particular samples or platforms and reducing the independence of findings across studies. The RDoC analysis was intended to propose exploratory conceptual links rather than establish validated correspondences. Future research could better incorporate expert consensus and user or patient interviews to understand the contextual and subjective meanings of digital behaviors and to examine their potential links to RDoC constructs further.

### Conclusions

Overall, existing studies have examined a diverse range of digital behavioral features. Still, substantial variation remains in how conceptually similar features are defined and operationalized, and no individual feature has shown a stable and consistently directed association across studies. Some studies reported only feature-set or multimodal model-level results, further limiting interpretation of specific digital behavioral features. The exploratory RDoC analysis further suggests that digital features may be better understood as observable traces of real-world behavior that can be used to generate hypotheses about underlying functional processes, rather than as direct proxies for specific psychological constructs. Future research should improve the transparency of feature definitions and result reporting, replicate findings across independent samples, and adopt construct-informed study designs to enhance the interpretability and reproducibility of digital phenotyping research in depression.

## Appendix

Multimedia Appendix 1 PRISMA-ScR checklist.

Multimedia Appendix 2 Search strategies.

Multimedia Appendix 3 Characteristics of the included studies.

Multimedia Appendix 4 Detailed digital behavioral features.

## Abbreviations

DSM-5: Diagnostic and Statistical Manual of Mental Disorders, Fifth Edition
DSM-IV-TR: Diagnostic and Statistical Manual of Mental Disorders, Fourth Edition, Text Revision
ICD-10: International Classification of Diseases, 10th Revision
NIMH: National Institute of Mental Health
PHQ-8: 8-item Patient Health Questionnaire
PHQ-9: 9-item Patient Health Questionnaire
PRISMA-ScR: Preferred Reporting Items for Systematic Reviews and Meta-Analyses Extension for Scoping Reviews
RDoC: research domain criteria

## Data Availability

All data generated or analyzed during this study are included in this published article and its supplementary information files.
