# Supplementary material for: Online Behavioral Digital Phenotyping for Depression: Scoping Review of Behavioral Features and Exploratory Assessment of Research Domain Criteria Construct Relevance
Source: J Med Internet Res. 2026 Oct 21;28:e101329. doi: 10.2196/101329 (PMC13735187; doi:10.2196/101329)
Supplement: Supplementary file 2 [file jmir_v28i1e101329_app2.docx]

**The complete search strategy**

| **Database** | **Search strategy** | **Result** |
| --- | --- | --- |
| PubMed | #1  "Depression"[MeSH Terms] OR "Depressive Disorder"[Title/Abstract] OR "Major Depressive Disorder"[Title/Abstract] OR "MDD"[Title/Abstract] OR "Persistent Depressive Disorder"[Title/Abstract] OR "Depressive Episode"[Title/Abstract] OR "Unipolar Depression"[Title/Abstract] OR "Depressive Syndrome"[Title/Abstract] OR "depress*"[Title/Abstract] 685,991  #2  "digital biomarker*"[Title/Abstract] OR "digital phenotyping"[Title/Abstract] OR "passive sensing"[Title/Abstract] OR "smartphone"[Title/Abstract] OR "wearable*"[Title/Abstract] OR "sensor*"[Title/Abstract] OR "web search"[Title/Abstract] OR "text message"[Title/Abstract] OR "keystroke dynamics"[Title/Abstract]  614,830  #3  "detect*"[Title/Abstract] OR "screen*"[Title/Abstract] OR "predict*"[Title/Abstract] OR "diagnosis"[Title/Abstract] OR "early diagnosis"[Title/Abstract] 7,754,850  #4 #1 AND #2 AND #3 3,659 | 3,659 2026/1/8 |
| Scopus | ( ( TITLE-ABS-KEY ( depression ) OR TITLE-ABS-KEY ( "Depressive Disorder" ) OR TITLE-ABS-KEY ( "Major Depressive Disorder" ) OR TITLE-ABS-KEY ( MDD ) OR TITLE-ABS-KEY ( "Persistent Depressive Disorder" ) OR TITLE-ABS-KEY ( "Depressive Episode" ) OR TITLE-ABS-KEY ( "Unipolar Depression" ) OR TITLE-ABS-KEY ( "Depressive Syndrome" ) OR TITLE-ABS-KEY ( depress* ) ) ) AND ( ( TITLE-ABS-KEY ( "digital biomarker*" ) OR TITLE-ABS-KEY ( "digital phenotyping" ) OR TITLE-ABS-KEY ( "passive sensing" ) OR TITLE-ABS-KEY ( smartphone ) OR TITLE-ABS-KEY ( wearable* ) OR TITLE-ABS-KEY ( sensor* ) OR TITLE-ABS-KEY ( "web search" ) OR TITLE-ABS-KEY ( "text message" ) OR TITLE-ABS-KEY ( "keystroke dynamics" ) ) ) AND ( ( TITLE-ABS-KEY ( detect* ) OR TITLE-ABS-KEY ( screen* ) OR TITLE-ABS-KEY ( predict* ) OR TITLE-ABS-KEY ( diagnosis ) OR TITLE-ABS-KEY ( early diagnosis ) ) ) | 8,579  2026/1/9 |
| Web of Science | #1  TS=(Depression) OR TS=("Depressive Disorder") OR TS=("Major Depressive Disorder") OR TS=(MDD) OR TS=("Persistent Depressive Disorder") OR TS=("Depressive Episode") OR TS=("Unipolar Depression") OR TS=("Depressive Syndrome") OR TS=(depress*) 577,666  #2  TS=("digital biomarker*") OR TS=("digital phenotyping") OR TS=("passive sensing") OR TS=(smartphone) OR TS=(wearable*) OR TS=(sensor*) OR TS=("web search") OR TS=("text message") OR TS=("keystroke dynamics") 1,163,724  #3  TS=(detect*) OR TS=(screen*) OR TS=(predict*) OR TS=(diagnosis) OR TS=(early diagnosis) 8,558,626  #4  #1 AND #2 AND #3 4,420 | 4,420  2026/1/9 |
| Embase | #1  'depression'/exp OR 'depressive disorder' OR 'major depressive disorder':ab,ti OR mdd:ab,ti OR 'persistent depressive disorder':ab,ti OR 'depressive episode':ab,ti OR 'unipolar depression':ab,ti OR 'depressive syndrome':ab,ti OR depress*:ab,ti 1,170,061  #2  'digital biomarker*':ab,ti OR 'digital phenotyping':ab,ti OR 'passive sensing':ab,ti OR smartphone:ab,ti OR wearable*:ab,ti OR sensor*:ab,ti OR 'web search':ab,ti OR 'text message':ab,ti OR 'keystroke dynamics':ab,ti 719,517  #3  detect*:ab,ti OR screen*:ab,ti OR predict*:ab,ti OR diagnosis:ab,ti OR 'early diagnosis':ab,ti 10,504,864  #1 AND #2 AND #3 7,479 | 7,479  2026/1/9 |
| PsycINFO | S1  SU (Depression) OR XB ("Depressive Disorder") OR XB ("Major Depressive Disorder") OR XB (MDD) OR XB ("Persistent Depressive Disorder") OR XB ("Depressive Episode") OR XB ("Unipolar Depression") OR XB ("Depressive Syndrome") OR XB (depress*) 406,931  S2  XB ("digital biomarker*") OR XB ("digital phenotyping") OR XB ("passive sensing") OR XB (smartphone) OR XB (wearable*) OR XB (sensor*) OR XB ("web search") OR XB ("text message") OR XB ("keystroke dynamics") 128,926  S3  XB (detect*) OR XB (screen*) OR XB (predict*) OR XB (diagnosis) OR XB ("early diagnosis") 1,010,703  S1 AND S2 AND S3 1,608 | 1608  2026/1/15 |
